# *N*-[2-(3,4-Dimeth­oxy­phen­yl)eth­yl]-*N*,4-dimethyl­benzene­sulfonamide

**DOI:** 10.1107/S1600536811029746

**Published:** 2011-07-30

**Authors:** Jasmine P. Vennila, D. John Thiruvadigal, Helen P. Kavitha, G. Chakkaravarthi, V. Manivannan

**Affiliations:** a Department of Physics, Panimalar Institute of Technology, Chennai 602 103, India; b Department of Physics, SRM University, Kattankulathur Campus, Chennai, India; c Department of Chemistry, SRM University, Ramapuram Campus, Chennai 600 089, India; d Department of Physics, CPCL Polytechnic College, Chennai 600 068, India; e Department of Research and Development, PRIST University, Vallam, Thanjavur 613 403, Tamil Nadu, India

## Abstract

In the title compound, C_18_H_23_NO_4_S, the dihedral angle between the two aromatic rings is 29.14 (7)°. The S atom has a distorted tetra­hedral geometry [106.15 (9)–119.54 (10)°]. The crystal structure exhibits weak C—H⋯O and π–π inter­actions.

## Related literature

For the biological activity of sulfonamide derivatives, see: Chumakov *et al.* (2006 ▶); Kremer *et al.* (2006 ▶). For related structures, see: Khan *et al.* (2010 ▶); Sharif *et al.* (2010 ▶).
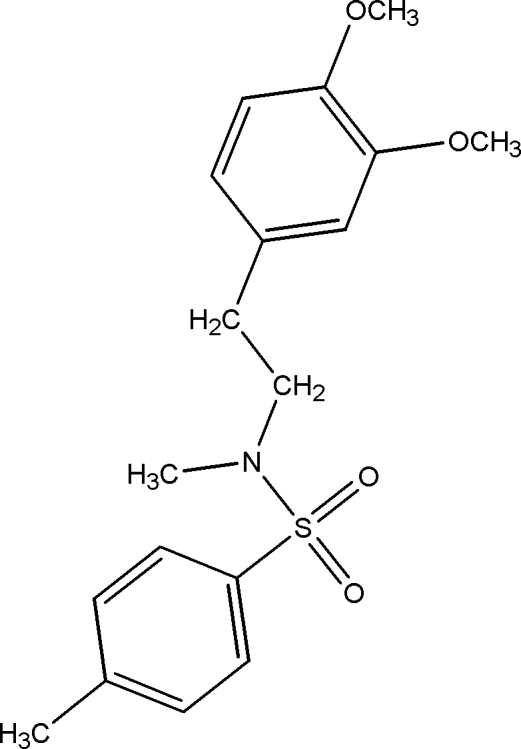


## Experimental

### 

#### Crystal data

C_18_H_23_NO_4_S
*M* *_r_* = 349.43
Monoclinic, 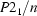

*a* = 5.7814 (4) Å
*b* = 13.9861 (12) Å
*c* = 21.9791 (18) Å
β = 92.949 (4)°
*V* = 1774.9 (2) Å^3^
*Z* = 4
Mo *K*α radiation
μ = 0.20 mm^−1^
*T* = 295 K
0.30 × 0.24 × 0.20 mm

#### Data collection

Bruker Kappa APEXII diffractometer
Absorption correction: multi-scan (*SADABS*; Sheldrick, 1996 ▶) *T*_min_ = 0.942, *T*_max_ = 0.960
18724 measured reflections
3343 independent reflections
2614 reflections with *I* > 2σ(*I*)
*R*_int_ = 0.036

#### Refinement

*R*[*F* ^2^ > 2σ(*F* ^2^)] = 0.040
*wR*(*F* ^2^) = 0.110
*S* = 1.04
3343 reflections
221 parameters
H-atom parameters constrained
Δρ_max_ = 0.19 e Å^−3^
Δρ_min_ = −0.30 e Å^−3^

### 

Data collection: *APEX2* (Bruker, 2004 ▶); cell refinement: *SAINT* (Bruker, 2004 ▶); data reduction: *SAINT*; program(s) used to solve structure: *SHELXS97* (Sheldrick, 2008 ▶); program(s) used to refine structure: *SHELXL97* (Sheldrick, 2008 ▶); molecular graphics: *PLATON* (Spek, 2009 ▶); software used to prepare material for publication: *SHELXL97*.

## Supplementary Material

Crystal structure: contains datablock(s) global, I. DOI: 10.1107/S1600536811029746/bt5588sup1.cif

Structure factors: contains datablock(s) I. DOI: 10.1107/S1600536811029746/bt5588Isup2.hkl

Supplementary material file. DOI: 10.1107/S1600536811029746/bt5588Isup3.cml

Additional supplementary materials:  crystallographic information; 3D view; checkCIF report

## Figures and Tables

**Table 1:** Hydrogen-bond geometry (Å, °)

*D*—H⋯*A*	*D*—H	H⋯*A*	*D*⋯*A*	*D*—H⋯*A*
C12—H12⋯O1^i^	0.93	2.54	3.452 (2)	166
C18—H18*B*⋯O2^ii^	0.96	2.38	3.302 (3)	160

Symmetry codes: (i) 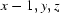; (ii) 
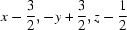
.

**Fig. 1.**
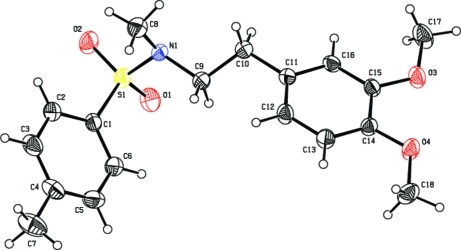
The molecular structure of (I), with atom labels and 30% probability displacement ellipsoids for non-H atoms.

**Fig. 2.**
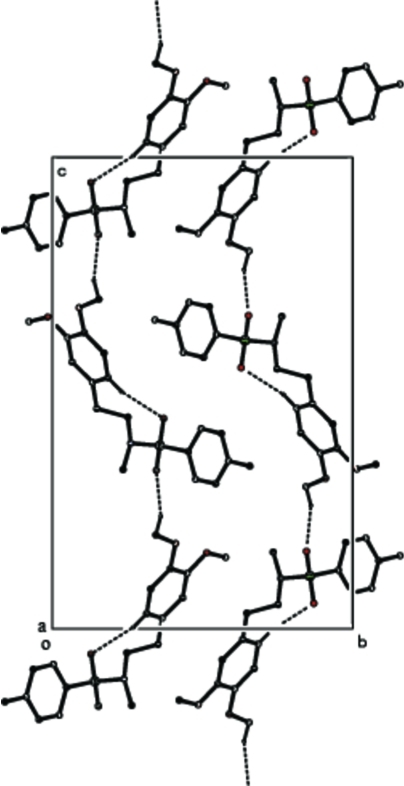
The packing of (I), viewed down the a axis. H-bonds are shown as dashed lines; H atoms not involved in hydrogen bonding have been omitted.

## supplementary crystallographic information

### Comment

Sulfonamide derivatives are extensively used in medicine as they possess a wide range of medicinal, pharmacological and antimicrobial properties (Chumakov *et al.*, 2006, Kremer *et al.*, 2006). We report the crystal structure of the titled compound (I) (Fig. 1).

In the title compound (I), the geometric pararameters are similar with the reported similar structures (Khan *et al.*, 2010; Sharif *et al.*, 2010). The S atom of the title molecule has a distorted tetrahedral geometry, with S1—O1 = 1.4210 (15), S1—O2 = 1.4195 (15), S1—N1 = 1.6391 (17), S1—C1 = 1.7538 (19) Å, O1—S1—O2 = 119.54 (10), O1—S1—N1 = 106.83 (9), O1—S1—C7 = 108.64 (9), O2—S1—N1 = 106.32 (9), O2—S1—C7 = 108.57 (9) and N1—S1—C7 = 106.15 (9)°.

The dihedral angle between the two  rings C1—C6 and C11—C16 is 29.14 (7)°. The crystal structure exhibits weak C—H···O (Fig.2 and Table 1) and π–π [*Cg*1···*Cg*2 (-*x*,2 - *y*,-*z*) distance of 5.2909 (13)Å and *Cg*2···*Cg*2 (-*x*,1 - *y*,-*z*) distance of 4.7146 (12) Å; *Cg*1 and *Cg*2 are the centroids of the rings C1—C6 and C11—C16, respectively] interactions.

### Experimental

2-(3,4-dimethoxyphenyl)-*N*-methyl ethanamine (51 mmol) was dissolved in dichloromethane (20 ml) in a round bottom flask. To this, added triethylamine (10.2 mmol) with stirring for 5 minutes. Then 4-methylbenzene-1-sulfonyl chloride (51 mmol) was added into the reaction mass and heated to 50 °C for 6 hrs. After cooling the reaction mixture to the normal temperature, it was added to water (20 ml). The aqueous layer was separated. The ethyl acetate layer was washed twice with 10% sodium chloride solution. The organic layer was dried over 2 g of anhydrous sodium sulfate and filtered. The filtrate was evaporated under vacuum to isolate the crude compound. Recrystallization of the compound using ethyl acetate and hexane mixture yielded the diffraction quality crystals.

### Refinement

All H atoms were positioned geometrically with C—H = 0.93–0.97 Å and allowed to ride on their parent atoms, with *U*_iso_(H) = 1.2 or 1.5Ueq(C).

### Crystal data

**Table:**

C_18_H_23_NO_4_S	*F*(000) = 744
*M**_r_* = 349.43	*D*_x_ = 1.308 Mg m^−^^3^
Monoclinic, *P*2_1_/*n*	Mo *K*α radiation, λ = 0.71073 Å
Hall symbol: -P 2yn	Cell parameters from 6145 reflections
*a* = 5.7814 (4) Å	θ = 2.4–25.5°
*b* = 13.9861 (12) Å	µ = 0.20 mm^−^^1^
*c* = 21.9791 (18) Å	*T* = 295 K
β = 92.949 (4)°	Block, colourless
*V* = 1774.9 (2) Å^3^	0.30 × 0.24 × 0.20 mm
*Z* = 4	

### Data collection

**Table:**

Bruker Kappa APEXII diffractometer	3343 independent reflections
Radiation source: fine-focus sealed tube	2614 reflections with *I* > 2σ(*I*)
graphite	*R*_int_ = 0.036
ω and φ scans	θ_max_ = 25.8°, θ_min_ = 2.4°
Absorption correction: multi-scan (*SADABS*; Sheldrick, 1996)	*h* = −4→7
*T*_min_ = 0.942, *T*_max_ = 0.960	*k* = −16→17
18724 measured reflections	*l* = −26→26

### Refinement

**Table:**

Refinement on *F*^2^	Primary atom site location: structure-invariant direct methods
Least-squares matrix: full	Secondary atom site location: difference Fourier map
*R*[*F*^2^ > 2σ(*F*^2^)] = 0.040	Hydrogen site location: inferred from neighbouring sites
*wR*(*F*^2^) = 0.110	H-atom parameters constrained
*S* = 1.04	*w* = 1/[σ^2^(*F*_o_^2^) + (0.0471*P*)^2^ + 0.6472*P*] where *P* = (*F*_o_^2^ + 2*F*_c_^2^)/3
3343 reflections	(Δ/σ)_max_ < 0.001
221 parameters	Δρ_max_ = 0.19 e Å^−^^3^
0 restraints	Δρ_min_ = −0.30 e Å^−^^3^

### Special details

**Table:**

Geometry. All e.s.d.'s (except the e.s.d. in the dihedral angle between two l.s. planes) are estimated using the full covariance matrix. The cell e.s.d.'s are taken into account individually in the estimation of e.s.d.'s in distances, angles and torsion angles; correlations between e.s.d.'s in cell parameters are only used when they are defined by crystal symmetry. An approximate (isotropic) treatment of cell e.s.d.'s is used for estimating e.s.d.'s involving l.s. planes.

### Fractional atomic coordinates and isotropic or equivalent isotropic displacement parameters (Å^2^)

**Table:**

	*x*	*y*	*z*	*U*_iso_*/*U*_eq_	
S1	0.58829 (8)	0.85799 (4)	0.11159 (2)	0.04660 (17)	
O1	0.6833 (3)	0.87108 (11)	0.05383 (7)	0.0609 (4)	
O2	0.7388 (3)	0.84655 (12)	0.16418 (7)	0.0663 (5)	
O3	0.0375 (3)	0.48564 (11)	−0.16121 (7)	0.0607 (4)	
O4	−0.3215 (2)	0.58964 (12)	−0.18094 (6)	0.0612 (4)	
N1	0.4284 (3)	0.76123 (11)	0.10662 (7)	0.0430 (4)	
C1	0.3980 (3)	0.95272 (13)	0.12431 (8)	0.0425 (4)	
C2	0.3502 (4)	0.97724 (15)	0.18308 (9)	0.0537 (5)	
H2	0.4308	0.9487	0.2160	0.064*	
C3	0.1823 (4)	1.04423 (16)	0.19236 (11)	0.0628 (6)	
H3	0.1501	1.0610	0.2320	0.075*	
C4	0.0602 (4)	1.08733 (15)	0.14425 (11)	0.0597 (6)	
C5	0.1148 (4)	1.06328 (16)	0.08611 (11)	0.0615 (6)	
H5	0.0368	1.0929	0.0532	0.074*	
C6	0.2822 (4)	0.99631 (15)	0.07548 (9)	0.0522 (5)	
H6	0.3167	0.9807	0.0358	0.063*	
C7	−0.1298 (5)	1.15787 (19)	0.15501 (15)	0.0872 (9)	
H7A	−0.0652	1.2209	0.1591	0.131*	
H7B	−0.2038	1.1412	0.1916	0.131*	
H7C	−0.2416	1.1565	0.1212	0.131*	
C8	0.3156 (4)	0.73564 (17)	0.16243 (10)	0.0587 (6)	
H8A	0.1778	0.7732	0.1656	0.088*	
H8B	0.4194	0.7478	0.1971	0.088*	
H8C	0.2755	0.6690	0.1613	0.088*	
C9	0.2771 (4)	0.75162 (14)	0.05110 (9)	0.0492 (5)	
H9A	0.3440	0.7863	0.0181	0.059*	
H9B	0.1272	0.7796	0.0580	0.059*	
C10	0.2458 (4)	0.64822 (14)	0.03316 (9)	0.0497 (5)	
H10A	0.3961	0.6211	0.0256	0.060*	
H10B	0.1841	0.6135	0.0669	0.060*	
C11	0.0868 (3)	0.63423 (13)	−0.02258 (8)	0.0408 (4)	
C12	−0.1148 (3)	0.68548 (15)	−0.03237 (9)	0.0489 (5)	
H12	−0.1572	0.7298	−0.0034	0.059*	
C13	−0.2558 (3)	0.67224 (15)	−0.08459 (9)	0.0498 (5)	
H13	−0.3904	0.7082	−0.0905	0.060*	
C14	−0.1991 (3)	0.60677 (14)	−0.12757 (8)	0.0420 (4)	
C15	0.0004 (3)	0.55133 (13)	−0.11718 (8)	0.0416 (4)	
C16	0.1404 (3)	0.56603 (13)	−0.06575 (9)	0.0431 (5)	
H16	0.2742	0.5296	−0.0596	0.052*	
C17	0.2289 (4)	0.42275 (18)	−0.15152 (13)	0.0742 (7)	
H17A	0.3703	0.4588	−0.1508	0.111*	
H17B	0.2287	0.3767	−0.1839	0.111*	
H17C	0.2169	0.3903	−0.1133	0.111*	
C18	−0.4992 (4)	0.65515 (19)	−0.19872 (11)	0.0662 (7)	
H18A	−0.6191	0.6526	−0.1701	0.099*	
H18B	−0.5626	0.6386	−0.2386	0.099*	
H18C	−0.4363	0.7186	−0.1995	0.099*	

### Atomic displacement parameters (Å^2^)

**Table:**

	*U*^11^	*U*^22^	*U*^33^	*U*^12^	*U*^13^	*U*^23^
S1	0.0386 (3)	0.0522 (3)	0.0483 (3)	0.0061 (2)	−0.0042 (2)	−0.0112 (2)
O1	0.0549 (9)	0.0663 (10)	0.0630 (10)	0.0020 (7)	0.0177 (7)	−0.0123 (8)
O2	0.0512 (8)	0.0763 (11)	0.0685 (10)	0.0111 (8)	−0.0249 (7)	−0.0178 (8)
O3	0.0600 (9)	0.0594 (9)	0.0614 (9)	0.0207 (7)	−0.0094 (7)	−0.0241 (7)
O4	0.0541 (8)	0.0736 (11)	0.0538 (9)	0.0195 (7)	−0.0170 (7)	−0.0185 (8)
N1	0.0453 (9)	0.0435 (9)	0.0394 (9)	0.0073 (7)	−0.0075 (7)	−0.0039 (7)
C1	0.0457 (10)	0.0391 (10)	0.0425 (11)	0.0008 (8)	0.0010 (8)	−0.0059 (8)
C2	0.0714 (14)	0.0478 (12)	0.0420 (12)	0.0067 (10)	0.0031 (10)	−0.0046 (9)
C3	0.0869 (17)	0.0490 (13)	0.0548 (14)	0.0079 (12)	0.0246 (12)	−0.0054 (11)
C4	0.0648 (14)	0.0407 (12)	0.0757 (16)	0.0065 (10)	0.0224 (12)	0.0010 (11)
C5	0.0687 (14)	0.0523 (13)	0.0631 (14)	0.0140 (11)	−0.0003 (11)	0.0081 (11)
C6	0.0634 (13)	0.0507 (12)	0.0426 (11)	0.0062 (10)	0.0041 (9)	−0.0023 (9)
C7	0.0838 (18)	0.0608 (16)	0.120 (2)	0.0226 (14)	0.0352 (17)	0.0052 (16)
C8	0.0647 (14)	0.0592 (14)	0.0520 (13)	0.0055 (11)	0.0017 (10)	−0.0009 (11)
C9	0.0508 (11)	0.0448 (11)	0.0501 (12)	0.0040 (9)	−0.0139 (9)	−0.0035 (9)
C10	0.0555 (12)	0.0445 (12)	0.0480 (12)	0.0055 (9)	−0.0088 (9)	−0.0028 (9)
C11	0.0425 (10)	0.0379 (10)	0.0416 (11)	0.0011 (8)	−0.0021 (8)	−0.0006 (8)
C12	0.0471 (11)	0.0535 (12)	0.0459 (11)	0.0088 (9)	0.0014 (9)	−0.0142 (9)
C13	0.0390 (10)	0.0563 (12)	0.0536 (12)	0.0140 (9)	−0.0031 (9)	−0.0107 (10)
C14	0.0378 (9)	0.0457 (11)	0.0420 (11)	0.0025 (8)	−0.0031 (8)	−0.0035 (9)
C15	0.0424 (10)	0.0368 (10)	0.0453 (11)	0.0031 (8)	0.0012 (8)	−0.0068 (8)
C16	0.0407 (10)	0.0354 (10)	0.0525 (12)	0.0076 (8)	−0.0036 (8)	−0.0023 (9)
C17	0.0615 (14)	0.0631 (16)	0.0972 (19)	0.0222 (12)	−0.0040 (13)	−0.0318 (14)
C18	0.0567 (13)	0.0845 (18)	0.0556 (14)	0.0189 (12)	−0.0147 (11)	−0.0004 (12)

### Geometric parameters (Å, °)

**Table:**

S1—O2	1.4195 (15)	C8—H8A	0.9600
S1—O1	1.4210 (15)	C8—H8B	0.9600
S1—N1	1.6391 (17)	C8—H8C	0.9600
S1—C1	1.7538 (19)	C9—C10	1.507 (3)
O3—C15	1.359 (2)	C9—H9A	0.9700
O3—C17	1.421 (3)	C9—H9B	0.9700
O4—C14	1.360 (2)	C10—C11	1.506 (3)
O4—C18	1.417 (2)	C10—H10A	0.9700
N1—C8	1.463 (3)	C10—H10B	0.9700
N1—C9	1.471 (2)	C11—C12	1.376 (3)
C1—C6	1.378 (3)	C11—C16	1.392 (3)
C1—C2	1.378 (3)	C12—C13	1.386 (3)
C2—C3	1.372 (3)	C12—H12	0.9300
C2—H2	0.9300	C13—C14	1.367 (3)
C3—C4	1.380 (3)	C13—H13	0.9300
C3—H3	0.9300	C14—C15	1.399 (3)
C4—C5	1.374 (3)	C15—C16	1.372 (2)
C4—C7	1.504 (3)	C16—H16	0.9300
C5—C6	1.375 (3)	C17—H17A	0.9600
C5—H5	0.9300	C17—H17B	0.9600
C6—H6	0.9300	C17—H17C	0.9600
C7—H7A	0.9600	C18—H18A	0.9600
C7—H7B	0.9600	C18—H18B	0.9600
C7—H7C	0.9600	C18—H18C	0.9600
			
O2—S1—O1	119.54 (10)	N1—C9—H9A	109.4
O2—S1—N1	106.32 (9)	C10—C9—H9A	109.4
O1—S1—N1	106.83 (9)	N1—C9—H9B	109.4
O2—S1—C1	108.57 (9)	C10—C9—H9B	109.4
O1—S1—C1	108.64 (9)	H9A—C9—H9B	108.0
N1—S1—C1	106.15 (9)	C11—C10—C9	113.42 (16)
C15—O3—C17	117.52 (16)	C11—C10—H10A	108.9
C14—O4—C18	117.51 (16)	C9—C10—H10A	108.9
C8—N1—C9	113.66 (16)	C11—C10—H10B	108.9
C8—N1—S1	114.86 (13)	C9—C10—H10B	108.9
C9—N1—S1	116.11 (13)	H10A—C10—H10B	107.7
C6—C1—C2	120.49 (18)	C12—C11—C16	117.84 (17)
C6—C1—S1	119.59 (15)	C12—C11—C10	122.44 (17)
C2—C1—S1	119.67 (15)	C16—C11—C10	119.71 (16)
C3—C2—C1	119.1 (2)	C11—C12—C13	121.17 (18)
C3—C2—H2	120.4	C11—C12—H12	119.4
C1—C2—H2	120.4	C13—C12—H12	119.4
C2—C3—C4	121.5 (2)	C14—C13—C12	120.65 (18)
C2—C3—H3	119.2	C14—C13—H13	119.7
C4—C3—H3	119.2	C12—C13—H13	119.7
C5—C4—C3	118.2 (2)	O4—C14—C13	125.54 (17)
C5—C4—C7	120.8 (2)	O4—C14—C15	115.54 (16)
C3—C4—C7	121.0 (2)	C13—C14—C15	118.91 (17)
C4—C5—C6	121.5 (2)	O3—C15—C16	125.43 (17)
C4—C5—H5	119.3	O3—C15—C14	114.69 (16)
C6—C5—H5	119.3	C16—C15—C14	119.88 (17)
C5—C6—C1	119.15 (19)	C15—C16—C11	121.46 (17)
C5—C6—H6	120.4	C15—C16—H16	119.3
C1—C6—H6	120.4	C11—C16—H16	119.3
C4—C7—H7A	109.5	O3—C17—H17A	109.5
C4—C7—H7B	109.5	O3—C17—H17B	109.5
H7A—C7—H7B	109.5	H17A—C17—H17B	109.5
C4—C7—H7C	109.5	O3—C17—H17C	109.5
H7A—C7—H7C	109.5	H17A—C17—H17C	109.5
H7B—C7—H7C	109.5	H17B—C17—H17C	109.5
N1—C8—H8A	109.5	O4—C18—H18A	109.5
N1—C8—H8B	109.5	O4—C18—H18B	109.5
H8A—C8—H8B	109.5	H18A—C18—H18B	109.5
N1—C8—H8C	109.5	O4—C18—H18C	109.5
H8A—C8—H8C	109.5	H18A—C18—H18C	109.5
H8B—C8—H8C	109.5	H18B—C18—H18C	109.5
N1—C9—C10	111.29 (16)		
			
O2—S1—N1—C8	51.06 (16)	C8—N1—C9—C10	−75.5 (2)
O1—S1—N1—C8	179.76 (14)	S1—N1—C9—C10	147.97 (15)
C1—S1—N1—C8	−64.43 (15)	N1—C9—C10—C11	178.43 (16)
O2—S1—N1—C9	−172.91 (13)	C9—C10—C11—C12	−41.2 (3)
O1—S1—N1—C9	−44.21 (15)	C9—C10—C11—C16	140.1 (2)
C1—S1—N1—C9	71.59 (15)	C16—C11—C12—C13	−2.3 (3)
O2—S1—C1—C6	161.10 (17)	C10—C11—C12—C13	178.9 (2)
O1—S1—C1—C6	29.63 (19)	C11—C12—C13—C14	0.8 (3)
N1—S1—C1—C6	−84.94 (18)	C18—O4—C14—C13	11.4 (3)
O2—S1—C1—C2	−24.6 (2)	C18—O4—C14—C15	−168.70 (19)
O1—S1—C1—C2	−156.06 (17)	C12—C13—C14—O4	−178.3 (2)
N1—S1—C1—C2	89.36 (18)	C12—C13—C14—C15	1.8 (3)
C6—C1—C2—C3	1.4 (3)	C17—O3—C15—C16	4.2 (3)
S1—C1—C2—C3	−172.90 (17)	C17—O3—C15—C14	−176.0 (2)
C1—C2—C3—C4	0.1 (4)	O4—C14—C15—O3	−2.4 (3)
C2—C3—C4—C5	−1.6 (4)	C13—C14—C15—O3	177.47 (19)
C2—C3—C4—C7	177.6 (2)	O4—C14—C15—C16	177.38 (18)
C3—C4—C5—C6	1.7 (4)	C13—C14—C15—C16	−2.7 (3)
C7—C4—C5—C6	−177.6 (2)	O3—C15—C16—C11	−179.08 (18)
C4—C5—C6—C1	−0.2 (3)	C14—C15—C16—C11	1.2 (3)
C2—C1—C6—C5	−1.3 (3)	C12—C11—C16—C15	1.4 (3)
S1—C1—C6—C5	172.95 (17)	C10—C11—C16—C15	−179.82 (18)

### Hydrogen-bond geometry (Å, °)

**Table:**

*D*—H···*A*	*D*—H	H···*A*	*D*···*A*	*D*—H···*A*
C2—H2···O2	0.93	2.59	2.942 (3)	103
C8—H8B···O2	0.96	2.44	2.895 (3)	109
C9—H9A···O1	0.97	2.39	2.880 (3)	111
C12—H12···O1^i^	0.93	2.54	3.452 (2)	166
C18—H18B···O2^ii^	0.96	2.38	3.302 (3)	160

Symmetry codes: (i) *x*−1, *y*, *z*; (ii) *x*−3/2, −*y*+3/2, *z*−1/2.
